# COVID-19 in the operating room: a review of evolving safety protocols

**DOI:** 10.1186/s13037-020-00254-6

**Published:** 2020-07-20

**Authors:** Lakshmanan Prakash, Shabir Ahmed Dhar, Muzaffar Mushtaq

**Affiliations:** 1 Institute of Special Orthopaedics, Palakkad, Kerala India; 2 SKIMS MC Bemina, Srinagar, Kashmir India

**Keywords:** COVID-19, Coronavirus, Surgeon, Health care workers, Protection, Orthopaedic surgery

## Abstract

**Background:**

The COVID-19 pandemic has already infected more than 3 million people across the world. As the healthworkers man the frontlines, the best practices model is continuously evolving as literature concerning the Coronavirus develops.

**Methods:**

A systematic review of the available literature was performed using the keyword terms “COVID-19”, “Coronavirus”, “surgeon”, “health-care workers”, “protection” and “Orthopaedic Surgery”. All peer-reviewed articles we could find were considered. Randomized controlled trials (RCTs), prospective trials and retrospective studies, as well as reviews and case reports, were included in this systematic review.

**Results:**

Even though surgical specialties including orthopedics are on the relative sidelines of the management of this pandemic but best practices models are inevitably developed for surgical specialties. The algorithm of postpone, delay, and operate only when life-threatening conditions exist is going to be useful up to a point.

**Conclusion:**

The surgical staff needs to keep abreast of the latest literature concerning safety measures to be taken during surgical procedures. Review articles can go some distance in helping in this educational process. This knowledge must evolve as new information comes to light.

## Introduction

The COVID 19 was labeled as a Pandemic on 11 March 2020 [1]. At the time of writing nearly 4 million people have been affected. The clinical spectrum of this disease is known to be very heterogeneous [2]. As the number of cases increases worldwide, the possibility of having to operate cases with the coronavirus infection is increasing. This will happen despite the current recommendations of operating only the emergent cases [3]. Cheney C in his write up on March 27 mentions that according to the guidelines of the centers for disease control and prevention elective surgery during the pandemic should be delayed [4]. These recommendations were made with the foresight of infrastructure and staff shortages. However, as the pandemic progresses two things have to be kept in mind by the health care authorities [1]. The increasing likelihood of COVID positive emergency cases presenting for surgical intervention.

The inevitability of having to restart elective procedures at some point in time. This is likely to happen whilst the pandemic is still around in some form. With the proven contagious properties of the virus and the relatively newer concept of the viral load, surgical safety measures need to be discussed as extensively as possible [2]. Gleaning from trauma literature it is seen that there is an increased likelihood of contracting COVID-19 in hospitals. Thousands of healthcare providers have been infected with COVID-19 despite their adherence to infection control measures [5].

This paper attempts to look at the current relatively scarce literature and answer some questions about the readiness and methods required for conducting safe surgery especially orthopedic intervention during the COVID-19 pandemic. The evolving literature must be published and read worldwide as COVID-19 is an occupational hazard to surgeons, health care workers, and their families.

## Material and method

A systematic review of the available literature was performed using the keyword terms “COVID-19”, “Coronavirus”, “surgeon”, “health-care workers”, “protection” and “Orthopaedic Surgery”. All peer-reviewed articles we could find were considered. Randomized controlled trials (RCTs), prospective trials and retrospective studies, as well as reviews and case reports, were included in this systematic review.

## Discussion

The current COVID-19 pandemic underlines the importance of careful and sensible utilization of financial and human resources. Preserving manpower is vital. A definite attempt should be made to minimize infection amongst surgeons and specialized professionals. While it is true that the surgical specialists are not at the forefront of managing the pandemic but two points have to be kept in mind vis a vis these specialists [6].

1. The likelihood of getting infected in the confines of the operation theatres is disproportionately high.
2. The training period of a surgeon is quite long. Replacing the surgeon is not a straightforward task.

The pandemic preparedness and literature evolution have mainly been on the Personal Protective Equipment (PPE) and Intensive Care Unit (ICU) areas. Not much has been written on the risks involved, methods and precautions required for an orthopedic surgeon and his operating room personnel whilst carrying out surgical procedures within the theatre.

Mild cases of COVID-19 have been found to have an early viral clearance, with 90% of these patients repeatedly testing negative on RT-PCR by day 10 post-onset. By contrast, all severe cases still tested positive at or beyond day 10 post-onset. Overall, our data indicate that, similar to SARS in 2002–03, patients with severe COVID-19 tend to have a high viral load and a long virus-shedding period. This finding suggests that the viral load of SARS-CoV-2 might be a useful marker for assessing disease severity and prognosis [2]. This concept of viral load is especially important for the operating surgeon.

Coccolini et al. believe that all known or suspected COVID-19 positive patients requiring surgical intervention must be treated as positive until proven otherwise to minimize infection spread [6]. However, it is preferable to order immediate SARS-CoV-2RT-PCR assay if a patient is being admitted and especially before surgery and possible intubation.

The surgeon also has to factor in staff sickness, reduced supply of surgical materials, alternate use of surgical facilities, and relatively lower availability of anaesthesiologists because of their additional intensive care loads [1].

Lei et al. studied 34 patients who underwent elective surgeries during the incubation period of COVID-19 at 3 hospitals. All their patients developed COVID-19 pneumonia shortly after surgery. 44.1% of patients needed intensive care postoperatively and the mortality rate was 20.5% [7]. Five of these surgeries were conducted in orthopedic specialist areas. The correlation is explained by the probable lowering of cell-mediated immunity after surgery which is vital for defense against viral infections [8]. Similar to the Middle East Respiratory Syndrome Coronavirus (MERS-CoV) infection, the Severe acute respiratory syndrome-related coronavirus (SARS-CoV) infected lung could induce and increase the amount of macrophage and neutrophil infiltration and increase the levels of pro-inflammatory cytokines and chemokines [9–11].

From the literature mentioned, it is clear that a COVID-19 patient undergoing surgery is at a higher risk of complications. Even though scientific literature on the surgeons getting affected during surgery is not available, it is important to view the media reports with more seriousness than usual [12].

### When to operate?

The SAGES and EAES recommendations regarding surgical response to COVID-19 mention that services should be rationed [13]. They have included the following points, amongst others, in the rationing

1. All elective surgical and endoscopic cases should be postponed at the current time.
2. All non-essential hospital or office staff should be allowed to stay home and telework.

The procedural considerations laid down in the same paper include

1. It is strongly recommended however, that consideration be given to the possibility of viral contamination to staff during surgery either open, laparoscopic, or robotic and that protective measures are strictly employed for OR staff safety and to maintain a functioning workforce.
2. For MIS procedures, the use of devices to filter released CO2 for aerosolized particles should be strongly considered.
3. There may be an enhanced risk of viral exposure to proceduralists.

The surgical team needs to be updated as to the latest protocols being used to ensure increased safety within the operation theatres to prevent the spread of the coronavirus outside the theatre and disease amongst the theatre personnel.

Philip F. Stahel published an editorial in which he divided elective procedures into “essential”, which bear an increased risk of adverse outcomes if surgery is delayed indefinitely, “non-essential” or “discretionary”, in which the results are not time-sensitive to surgery and “equivocal” which don’t fall clearly into one or the other category.

**Fig. 1:**
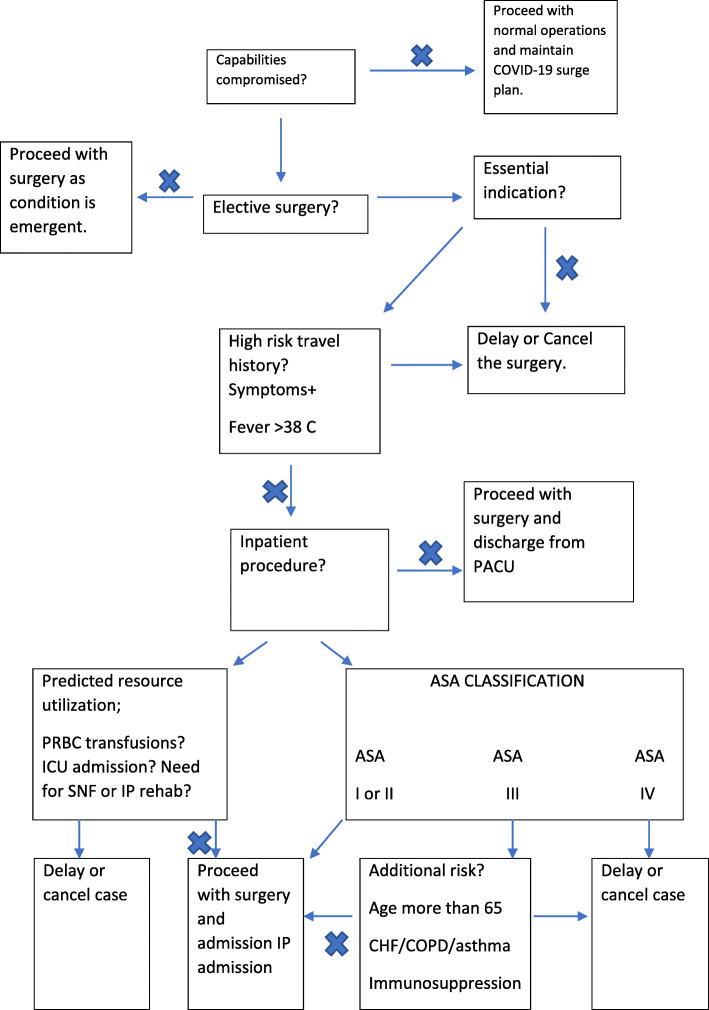
The decision-making algorithm for risk-stratification of elective surgical procedures. It is based on the underlying surgical indication and predicted resource utilization during the current COVID-19 pandemic [14]. Abbreviations: ASA, American Society of Anesthesiologists; CHF, chronic heart failure; COPD, chronic obstructive pulmonary disease; COVID, corona virus disease; ICU, intensive care unit; IP, inpatient; PACU, post-anesthesia care unit; PRBC, packed red blood cells; SNF, skilled nursing facility

He proposed an decision-making algorithm (Fig. 1) for deciding whether and when to proceed with an elective surgery, based on surgical indications and predicted requirement of critical resources, including blood product transfusions, estimated length of hospital stay, and the possible requirement for post-operative ventilation and ICU care [14].

### Where to operate?

Ti et al. have written that a relatively isolated theater with separate access and a negative pressure environment should be designated for such patients [15]. The negative pressure method is restricted in the anteroom and the induction room. The scrub area and the main operating room have positive pressures. The main operating room should have more than 25 air exchange cycles per hour. According to them understanding the airflow within the operation theatre is crucial to minimizing the risk of infection. Operating rooms are usually designed to have positive pressure to prevent intraoperative contamination. Coronavirus is 125 nm in diameter and a high proportion of particles [up to 100%] are captured by high-efficiency particulate air (HEPA) filters. This may be combined with the aforementioned high-frequency air exchanges to reduce the chance of virus dissemination [1, 16, 17].

Pinto et al. recommend that the operative complex be divided into 5 zones as shown in Table 1. This ensures an orderly process reducing dissemination.

**Table 1:** Zones of the operation theatre

Zone 1: Entry dressing room, where the basic PPE is donned	
Zone 2: Anteroom, where the disinfection and surgical dressing take place	
Zone 3: OR (COVID-19 room)	
Zone 4: Exit room, where the PPE is removed	
Zone 5: Exit dressing room, where the staff showers	

A route to minimize exposure and contact between triage to induction room, OR and then to recovery rooms should be frequently cleaned and disinfected [18].

### The surgeon and the theatre staff

The surgeons scrubbing routine has to change when entering the corona designated theatres. In Zone 1 a disposable surgical scrub suit, surgical boots, waterproof boot and a waterproof apron should be donned. Surgical hand preparation should also be done with water and chlorhexidine gluconate [1]. The surgeon should use either N95 or FFP 2 masks as recommended by the centers of disease control and prevention. They are effective for viruses including the coronavirus [19]. Powered air-purifying respirators [PAPR] is preferred for longer operations. Double surgical masks should be avoided especially in aerosolized blood generating procedures (Table 2).

**Table 2:** Personal protection equipment [5]

Personal protection equipment	
FFP2 facial mask	
FFP3 facial mask (in case of maneuvers at high risk of generating aerosolized particles)	
Disposable long sleeve waterproof coats, gowns, or Tyvek suits	
Disposable double pair of nitrile gloves	
Protective goggles or visors	
Disposable head caps	
Disposable long shoe covers	
Alcoholic hand hygiene solution	

*FFP* Filtering face piece

Eye protection equipment is also important during aerosol-generating procedures [18]. Full face shield or goggles are recommended.

In zone 2 either a surgical spacesuit or the second layer of sterile protective garments should be used. A surgical shield is also desirable. An aqueous alcohol solution is used for scrubbing. The first pair of gloves are donned. This should be followed by a sterile surgical scrub suit and second pair of gloves [1].

Surgical gowns (AAMI) [association of the advancement of medical instrumentation] - Level III (typically those found in operating rooms) or Coveralls should be prioritized for surgical and aerosolized-blood generating procedures. Surgical caps should be used as per protocol, but surgical hood with ties should be used for the head and the neck for aerosol-generating procedures [18]. Shoes or booties should be fluid resistant and double high cuffed surgical gloves are preferable.

After the surgery the staff exits through zone 4 where doffing is done. in zone 5 the scrub suit is removed and bathing is done.

Strict and frequent screening of the segregated OR staff is mandatory. Members of the segregated or exposed staff should immediately report any signs of illness and must be taken off duty immediately. Besides, all contact events between patients and staff must be recorded so that contact tracing and infection control measures can be implemented quickly, in case any member of segregated staff tests positive.

### Anesthesia considerations

The most experienced anaesthesiologist should intubate the patients. Ti et al. also recommended that the same operation theatre be used along with the same anesthesia machine for COVID cases. A heat and moisture exchanger (HME) filter is used on the expiratory limb of the circuit. The soda-lime and filters are exchanged after each case. Disposable airway equipment is to be used. The airway should be secured with the method which has the highest chance of first-time success especially video-laryngoscope [20]. Airway manipulation, face mask ventilation, and open airway suction should be minimized. Bag mask ventilation should also be avoided. If a patient is transferred directly from the intensive care unit, a dedicated transport ventilator should be utilized. To reduce aerosolization risks, the gas flow should be turned off and the endotracheal tube clamped with forceps when switching from the portable device to the OR ventilator [15]. Regional anesthesia is preferable. Nasal Oxygen should be administered under the surgical mask. Antiemetics should be used to reduce post-operative retching.

If the patient is already in an ICU, Firstenberg et al. recommended intubation in the negative flow ICU prior to transport to the OR by the attending intensivist, while using appropriate precautions including N95 mask or PPE, gown, eye protection and hair cover, to avoid exposure to the anaesthesia team [21].

### How to transfer

The transfer from the ward to the OR will be done by the ward nurses in full personal protective equipment (PPE) (Table 2) including a well-fitting N95 mask, goggles, or face shield, splash-resistant gown, and boot covers. For patients coming from the ICU, a dedicated transport ventilator is used. To avoid aerosolization, the gas flow is turned off and the endotracheal tube clamped with forceps [15]. Fisrtenberg et al. used a portable travel ventilator with a High-Efficiency Particulate Air (HEPA) filter placed between the endotracheal tube and the circuit and a second HEPA filter between the circuit and ventilator. They advised that two members of hospital security escort the transporting team to ensure elevator availability, open doors, and to minimize the risk of accidental contact with others during the transport [21].

While Li TK et al. have recommended an operation theater at the corner of the operation theatre complex with separate access, Coccolini et al. recommend an OT closest to the entrance of the complex [5, 15]. This probably depends on the architecture of the theatre complex as Pinto et al. mention a satellite position of the operation theatre [1]. They also recommend a 5-room complex.

Transfer routes should be as short as possible and precisely planned, with the same transport personnel throughout the shifting process [6]. We feel that this shall again vary depending on theater design and layout.

### Intraoperative protocol

Air exchange cycles should be increased whenever possible to ≥ 25 exchanges/h between surgeries [22, 23]. Even though no data currently exist on COVID-19 viral load in bodily fluids or tissue samples, extreme care is mandatory. Surgeons and personnel not needed for intubation should remain outside the operating room until anesthesia induction and intubation are completed for patients with or suspected of having COVID-19 infection [24].

Orthopedic surgery offers specific challenges and difficulties. Hart mentions that with the asymptomatic patients being quite large, the operation theatre might be a viral lab in a wind tunnel. Writing about orthopedic surgery he mentions that power tools, hammers, and other instruments spread a lot of material around. Even though we do not know about the concentration of COVID19 in blood and muscle, research into the airborne transmission of SARS and MERS makes it plausible that transmission is likely [25]. A recent Canadian study described low-fidelity simulation training to evolve the modified PPE used for aerosol-generating procedures of suspected/confirmed COVID-19 patients and assess sites of contamination [26]. The Spread of the aerosolized respiratory secretions and contamination sites were visualized with a commercial powder product and ultraviolet light. They demonstrated a significant amount of contamination on the provider’s neck, the base of the wrist, and their lower pants and shoes. Aerosols have been shown to spread from 5 to 7 m during orthopedic surgery. Hip replacement surgery can cause a spread of aerosol from 8 to 9 m [27, 28].

Minimum personnel should be placed in the operating room. Firstenberg et al. kept two runners were outside of the OR. Only minimum required supplies were opened in the procedure room, and whenever required the runners fetched the additional supplies needed for the case which were placed on a cart in the containment room and transported into the OR only when the outside door was shut. No additional phones or breaks/changes in staff should be allowed [21].

A smoke evacuator should be used when electrocautery is to be used. Particles in surgical smoke have been demonstrated to contain a variety of toxic and virulent materials thought to be capable of infecting those who inhale them, with case reports of doctors contracting rare papillomavirus when surgical smoke exposure was suspected to be the source [29]. Hence electrosurgery should be minimized or excluded. Bulb syringes should be used for lavage.

In trauma and orthopedic surgical procedures, the use of power tools, such as electrocautery, bone saws, reamers, and drills, releases aerosols [15], increasing the risk of virus spread. As such, their use should be reduced to the minimum and the power settings should be as low as possible [30]. Some possible complications include the formation of a viral biofilm.

Disposable medical equipment should be used. A sharp injury should be avoided to the PPE. All body fluids, blood, secretions, pathological specimens should be disposed of in double bags that are sealed. Any specimens taken should be placed into a biohazard bag inside the OR and subsequently placed in another biohazard bag in the containment room before being sent to the lab being properly labeled, for example as “Covid positive” or “suspected covid” [21].

COVID-19 is presumed to spread directly via infectious respiratory droplets and close contact (since SARS-CoV-2 cannot survive without carrier) [12] However, these transmission modes do not explain all cases. Recent data has shown that COVID-19 might survive and be transmitted indirectly from virus contamination of common surfaces and objects after virus aerosolization in a confined space with infected individuals [31]. The incubation period for COVID-19 is approximately 4 days and studies suggest it may range anywhere from 2 to 14 days. Individuals with respiratory sickness should not be allowed to donate blood due to a lack of definitive evidence of blood transmission of COVID-19 [32–34]. Theoretically, viremia in patients with asymptomatic or confirmed COVID-19 patients could pose a risk of transmissibility to the orthopedic team during aerosolized-blood generating procedures.

The theatre is to be disinfected between surgeries. But the disinfecting personnel should enter the theatre only after enough air changes have occurred to remove infectious particles [18]. If possible, no other surgery should be carried out in the same OR for the day, and theatre disinfected with UV light. The instruments sent to the sterilization unit must be labeled and the staff in the unit must be made aware of the covid status of the case, and must handle the instruments while wearing a full PPE [21].

The risk in theatres might be more in resource-constrained settings. Jain et al. mention that the emergence of COVID-19 has impacted orthopedic surgery worldwide. India, with its large population and limited health resources, will be overwrought over the coming days due to the number of cases of critically ill patients with COVID-19 [35].

Guo X et al. showed that 26 orthopedic surgeons got infected with COVID-19 in 3 hospitals in Wuhan. This highlights the fact that despite being a surgical specialty, the risk is quite high [36].

It is important to understand that aerosols can be generated either by surgery or by the respiration of the patient within the theatre [37, 38]. ENT, Neurosurgery, and Opthalmology surgeons are at risk from both types of aerosols while orthopedic surgeons are exposed to high levels of surgical aerosol but a lower risk of respiratory aerosol.

### Recovery

Surgeries that take longer time are likely to have more postoperative complications in a routine setting. These complications must be differentiated from COVID 19. Surgeons, nurses, and medical staff share equal responsibility for postoperative management, particularly in monitoring the patients’ families and visitors to ensure strict adherence to the pandemic emergency system. When possible, it is important to limit visitors as much as possible. Most hospitals have recently discontinued visitation by anyone [18]. Lei et al. showed significantly higher COVID related complications in these cases [7].

Massey e al recommended additional measures including physical Distancing and use of emerging technologies such as inpatient telemedicine and online file sharing applications to enable orthopedic programs to still function, while attempting to protect medical staff and patients from COVID-19 spread [39].

Cohen et al. suggest championing an alternative solution whereby we as a medical community become proactive rather than reactive, adopting a conservative yet balanced plan to protect both the patient and the health-care team. When faced with a biologically plausible concern that could infer serious harm, we are obligated to act with an abundance of caution, examining and questioning our standard practices [40].

It is acknowledged that, during the coronavirus pandemic, surgeons and patients will have difficult choices to make about management options for a wide variety of injuries and urgent conditions. They will need to balance optimum treatment of a patient’s injury or condition against clinical safety and resources [41].

The orthopedic community is continuing to develop strategies to deliver a safe musculoskeletal skeletal service at this difficult time, while many members of the orthopedic workforce move to the front line [42].

In the end, we must admit that however thorough our search through the literature maybe, in the current trend of evolving guidelines and protocols about surgical care of covid-19 patients, any meticulously drafted treatise will quickly become obsolete if not updated along. Here we list a few resources that we find immensely helpful for surgeons and orthopedic surgeons to keep themselves aware of the best practice guidelines issued from time to time by these reputed public health organizations:

- National Institute for Health and Care Excellence (UK): https://www.nice.org.uk/guidance/health-and-social-care-delivery/surgical-care
- World Health Organization: https://www.who.int/surgery/publications/immesc_best_practice/en/
- Centre for Disease Control and Prevention: https://www.cdc.gov/coronavirus/2019-ncov/hcp/preparedness-checklists.html
- Royal College of Surgeons, England: https://www.rcseng.ac.uk/standards-and-research/standards-and-guidance/good-practice-guides/coronavirus/covid-19-good-practice-for-surgeons-and-surgical-teams/
- American College of Surgeons: https://www.facs.org/covid-19/checklist
- American Association of Orthopaedic Surgeons: https://www.aaos.org/about/covid-19-information-for-our-members/guidance-for-elective-surgery/
- AO foundation: https://www.aofoundation.org/what-we-do/covid-19-resources-for-surgeons

## Conclusion

The surgical staff needs to keep abreast of the latest literature concerning safety measures to be taken during surgical procedures. Review articles can go some distance in helping in this educational process. This knowledge must evolve as new information comes to light. Infection or death of sub-specialized staff must be minimized to preserve the ability to face surgical emergencies and associated activities that will continue to occur or perhaps increase during a mass casualty incident.

### Core tip

It is important for surgical specialists to stay up to date with the latest information concerning safety measures while conducting surgeries. The evolution of literature should be closely followed so that the best practices are instituted and upgraded.

## Abbreviations

RCT: Randomised controlled trial
COVID: Corona virus disease
PPE: Personal protection equipment
ICU: Intensive care unit
RT-PCR: Reverse transcription polymerase chain reaction
SARS: Severe acute respiratory syndrome
MERS: Middle east respiratory syndrome
CoV: Corona virus
SAGES: Society of American gastrointestinal and endoscopic surgeons
EAES: European association for Endoscopic Surgery
HEPA: High efficiency particulate air
FFP: Filtering face piece
PAPR: Powered air purifying respirator
AAMI: Association of the advancement of medical instruments
HME: Heat and moisture exchanger
PPE: Personal protective equipment
